# Smoking treatment optimisation in pharmacies (STOP): a cluster randomised pilot trial of a training intervention

**DOI:** 10.1186/s40814-016-0120-9

**Published:** 2017-01-10

**Authors:** V. W. Madurasinghe, Ratna Sohanpal, Wai James, Liz Steed, Sandra Eldridge, SJC Taylor, C. Griffiths, Robert Walton

**Affiliations:** 1 Centre for Primary Care and Public Health, Barts and The London School of Medicine and Dentistry, Yvonne Carter Building, 58 Turner Street, London, E1 2AB UK; 2 Pragmatic Clinical Trials Unit (PCTU), Centre for Primary Care and Public Health, Blizard Institute, Yvonne Carter Building, 58 Turner Street, London, E1 2AB UK

**Keywords:** Smoking cessation, Community pharmacies, Training intervention, RCT, Cluster randomised, Pilot trial

## Abstract

**Background:**

UK government policy aims to strengthen the role of community pharmacies in health promotion. Thus, we conducted feasibility studies for an intervention to enhance delivery of the NHS Smoking Cessation Service.

**Methods:**

The overall aims were to assess acceptability and feasibility of conducting the intervention in community pharmacies and piloting this with a cluster randomised trial. Specific objectives were (1) to estimate likely participation rates of pharmacies and stop smoking advisors, (2) to establish the potential impact of the training intervention on throughput and retention of smokers in smoking services, (3) to establish potential impact on smoking cessation outcomes, (4) to optimise logistics for conducting a cluster randomised trial in the next phase of the research programme and (5) to consider the feasibility of collecting pharmacy and service user data.

In this cluster randomised parallel group pilot trial, 12 community pharmacies in East London were allocated to intervention or usual practice using simple randomisation (allocation ratio 2:1). Data were analysed descriptively.

**Results:**

Twelve of 54 (22.2%, 95% CI 12.0% to 35.6%) pharmacies and 20 of 23 (87.0%, 95% CI 66.4% to 97.2%) advisors invited, agreed to participate. Over 5 months, 302 smokers in intervention pharmacies (mean per pharmacy 43.1, 95% CI: −4.3 to 90.5) and 319 in usual practice pharmacies (mean per pharmacy 79.8, 95% CI: 19.0 to 140.5) joined the service. 51 of 621 smokers (6.3% in intervention vs 10.0% in usual practice) consented to provide additional data on smoking cessation. 17 of 19 smokers that consented were retained at 4 weeks in intervention arm (89.5%, 95% CI: 66.9% to 98.7%) and 24 of 32 in usual practice (75.0%, 95% CI: 56.6% to 88.5%). 10 of 19 in the intervention arm (52.6%, 95% CI: 28.9% to 75.6%) stopped smoking compared to 7 of 32 in usual practice arm (21.9%, 95% CI: 9.3% to 40.0%).

The pilot was useful in providing insights on how best to conduct the definitive trial and shortcomings of our present logistical arrangements, including feasibility of collecting pharmacy and service user data.

**Conclusions:**

Recruitment rates show that the main trial is feasible, and the results suggest that the intervention may improve retention and quit rates in smoking cessation services. We gained insights on how best to conduct the definitive trial which will proceed as planned.

## Background

Shortly after publication of the UK Department of Health white paper *Smoking kills*, [1] government targets were introduced to reduce the prevalence of adult smoking, and these targets were subsequently incorporated into the National Health Service (NHS) plan [2], the Cancer plan, the Priorities and Planning framework and the white paper *Choosing health* [3]. NHS smoking cessation services were established in 2000, and further policy initiatives led to legislation which banned smoking in public places. Following these changes there was a reduction in adult smoking in England from 28% in 1998 to 22% in 2006 [4].

However, one in five adults continue to smoke in the UK, and rates have not declined as rapidly amongst those most disadvantaged in society who suffer the major burden of tobacco-related disease [4] [5–11]. Further developments in government policy over this period led to strengthening primary care delivery through community pharmacies and substantial expansion of the role of pharmacists in managing a wide range of medical conditions [12, 13]. Smoking cessation was one of the first new clinical tasks taken on by pharmacists. In 2014/15, around 48% (84961 of 450582) of all quit attempts in the NHS smoking cessation service in England were made in community pharmacies [14].

There is a particular need to provide smoking cessation services for people who find it difficult to access conventional healthcare; the NHS pharmacy network may provide a means to address this problem, [4, 15] and pharmacy staff have contact with people less likely to attend more formal healthcare settings [15]. Increasing recruitment to pharmacy smoking cessation services and boosting success rates could therefore have a major impact on tobacco use in the community and wider public health. However, there is little research evidence on which to base service development in community pharmacies, and the quit rates achieved in this setting lag behind those seen in specialist services [15]. Whilst current evidence suggests that smoking cessation interventions can be effective in UK pharmacies, there are few trials with validated end points [16–18].

The Smoking Treatment Optimisation in Pharmacies (STOP) research programme is a National Institute for Health Research (NIHR) funded study that seeks to optimise NHS smoking cessation services in community pharmacies by developing a simple educational and training intervention. The training sessions and associated tools were designed for stop smoking advisors to address beliefs and attitudes which might limit effectiveness of the service and to expand their repertoire of consultation and behaviour change skills.

The STOP research programme is divided into four phases:

1. systematic review of studies on changing health behaviours in community pharmacies to identify aspects of successful interventions that could be applied to smoking cessation;
2. qualitative studies in service users and smoking advisors in successful and unsuccessful smoking cessation consultations;
3. development of the training intervention and pilot trial;
4. a cluster randomised trial and parallel process evaluation with 1200 NHS Smoking cessation service users in 60 pharmacies in East London to evaluate effectiveness and cost-effectiveness.

Findings from the systematic review [19] and qualitative studies [20] were used to develop the intervention, using elements of social cognitive theory [21] and self-determination theory [22] and making use of Michie’s behaviour change wheel and the COM-B model to select appropriate behaviour change techniques [23].

The overall aims of the pilot study were to assess acceptability and feasibility of conducting the intervention in community pharmacies and piloting this with a cluster randomised trial.

Specific objectives were:

1. to estimate likely participation rates of pharmacies and stop smoking advisors;
2. to establish the potential impact of the training intervention on throughput and retention of smokers in smoking services;
3. to establish potential impact on smoking cessation outcomes;
4. to optimise logistics for conducting a cluster randomised trial in the next phase of the research programme;
5. to consider the feasibility of collecting pharmacy and service user data.

## Methods

### Trial design

In this cluster randomised parallel group pilot study, 12 community pharmacies (clusters) were allocated to the STOP intervention or usual practice (allocation ratio 2:1) using simple randomisation. The randomisation list was generated using Stata 12 software. An independent statistician (CC) who was not part of the STOP team generated and administered the randomisation list. W-YJ and RS, who were enrolling community pharmacies, emailed CC with information from the consented pharmacies for allocation to the STOP intervention or to usual practice.

Since our intervention involved providing group training to stop smoking advisors, it was not possible to blind either participating pharmacies or the advisors. Service users in both intervention and usual practice pharmacies were informed that their community pharmacy was taking part in a smoking cessation study. However, they were not aware of pharmacy allocation status. The trial statistician (VM) remained blind to allocation status until the analysis was complete.

### Participants

There were two groups of trial participants:

1. community pharmacies (unit of randomisation) and smoking cessation advisors working within those pharmacies;
2. NHS smoking cessation service users.

#### Inclusion and exclusion criteria

##### Community pharmacies

Community pharmacies providing the NHS smoking cessation service in Newham, Tower Hamlets and City and Hackney boroughs were eligible. Community pharmacies outside these boroughs, non-community pharmacies (e.g. in hospitals/clinics), site that lacked facilities for secure storage and transfer of data and sites where smoking cessation advisors did not have governance training were excluded.

##### Smoking cessation advisors

All advisors working in the pharmacies were eligible. Advisors could either be pharmacists who owned or managed the pharmacy, or they could be employed staff. To be eligible advisors must have successfully completed National Centre for Smoking Cessation and Training (NCSCT) training.

##### Service users

Any service user eligible for the NHS smoking cessation service was eligible for this study. The inclusion criteria were:

1. adults (aged 18 years or more) who are eligible for the NHS smoking cessation service.
2. all ethnic groups and genders.
3. all levels of health behaviour: e.g. light (<10 cigs/day), moderate (10–20), heavy (>20) smokers.
4. all types of tobacco smoking: e.g. cigarettes, cigars, pipe, roll-ups and hookah pipes.
5. smokers who have never tried to quit through the service or who quit and then restarted smoking, or failed to quit previously.

Non-smokers and smokers under the age of 18 years were excluded.

#### Participant recruitment

##### Pharmacies and smoking cessation advisors

Fifty-four pharmacies were invited by letter (including stamped addressed envelope for response) and 33 were followed up with one phone call from a researcher (W-Y J or RS). 14 of 21 pharmacies expressing an interest were visited. Recruitment was closed when the 12 pharmacies necessary for the pilot trial had agreed to participate.

All smoking advisors within the participating pharmacies were provided with a study information sheet in plain English for them to make an informed decision about their individual participation in the study. Before obtaining consent, researchers (W-YJ or RS) explained the study to each advisor and checked their understanding. Advisors were given at least 24 h to consider their decision. Formal consent was obtained before taking part in the study using the approved consent form.

##### Service users

Smokers were not directly recruited into the main study; data on all service users in participating pharmacies were collected anonymously from routine data returns. Those that joined the NHS smoking cessation service were invited to provide individual level data. Written individual consent was sought from service users for: (i) use of identifiable routine data in STOP analyses; (ii) provision of a saliva sample for nicotine metabolites and deoxyribonucleic acid (DNA) for estimation of nicotine metabolism (iii) to be approached for an interview at 12 weeks to explore their experience of the NHS smoking cessation service. If service users did not wish to complete some parts of the study, they were given the option to decline to participate in those particular aspect(s) (for example if the service user did not wish to provide a saliva sample, it was not obligatory do so). Initially, smoking cessation advisors consented the service users on their second visit, however based on advisor feedback, the process was changed to taking consent at the first visit. Consent for accessing and using anonymised data at the pharmacy level was considered to be given under *gatekeeper agreement* by the owner or manager of the pharmacy [24].

### STOP intervention

We developed a programme theory for the initial version of the intervention which was based on findings from our qualitative studies and literature reviews. We were guided by the COM-B model of behaviour change and drew upon social cognitive theory and self-determination theory to target capability and motivation, respectively. We considered that addressing pharmacy workers skills, attitudes and motivation towards behaviour change, through practice based training sessions would lead to more effective engagement of smokers in the smoking cessation service and better quit rates.

The intervention comprised two face-to-face sessions aimed at smoking cessation advisors, 2 weeks apart. Each session lasted 2.5 h and was delivered by a health psychologist (LS) and an experienced community pharmacist who was also a trainer and smoking cessation advisor (DA). We chose a venue commonly used for other pharmacy staff training courses. Advisors were also encouraged to refresh their existing NCSCT training between sessions (see details below). The STOP intervention training involved role-plays and training videos targeting engagement of smokers and optimising the delivery of smoking cessation counselling. Attendees were given training handouts and a desktop flip chart with accompanying notes to act as a prompter and reinforcer within the pharmacy context. These materials were developed by the study team to support the messages from the training sessions. The sessions were intended to complement the training from the NCSCT. A Facebook page was also developed to signpost training materials and other smoking cessation resources. Full details of the intervention and its development are published separately.

### Usual practice

In usual practice, advisors received no additional training to that routinely provided which is typically qualification under the NCSCT certification programme [25–27]. NCSCT training is a competence-based programme aiming to impart knowledge and skills in smoking cessation. NCSCT training is a competence- based programme aiming to impart knowledge and skills in smoking cessation with relatively greater focus on the pharmacological elements of quitting smoking through nicotine replacement therapy rather than consultation skills such as engagement of smokers as targeted in our intervention.

The NCSCT training includes:

1. *Level 1 training or Very Brief Advice in Smoking Cessation training*
  This online training enables introducing the idea of quitting with individuals and can be undertaken by any healthcare professionals e.g. doctors, nurses, pharmacists including staff who advise people on how to quit smoking.
2. *Level 2 training or Training and Assessment programme*
  This online and face-to-face training is for equipping healthcare professionals, who intend to become stop smoking advisors, with knowledge and skills to provide intensive one-to-one support in smoking cessation through delivery of the NHS smoking cessation service.

### Outcome measures used to address study objectives

1. Participation rates for pharmacies were defined as the proportion of pharmacies choosing to take part in the STOP pilot trial using the number of pharmacies approached as the denominator.
2. Participation rates for stop smoking advisors were defined as the proportion of advisors choosing to take part in the STOP pilot trial. The denominator was the total number of smoking advisors in participating pharmacies.
3. Number of *treated smokers* i.e. a measure of throughput in the service, where treated smoker was defined as a smoker who sets a firm quit date and undergoes at least one treatment session on or prior to the quit date. Smokers who attend an assessment session but fail to attend thereafter did not meet the criterion for a treated smoker neither did smokers who had already stopped smoking at the time they first come to the attention of the service.
4. Retention in the service measured by subtracting the number of users *lost to follow up at 4 weeks* from the number of treated smokers. A treated smoker was counted as lost to follow up at 4 weeks (LFU4W) if, on attempting to determine the 4-week quitter status s/he could not be contacted using standard pharmacy procedures. Pharmacy staff were encouraged to validate the quitters by exhaled carbon monoxide (CO) in at least 85% of cases [15]. However, the proportion for which follow-up is attempted and the procedures used for following up may vary between pharmacies.
5. Carbon monoxide (CO) verified 4-week quit rate: a smoker is counted as a *CO-verified 4-week quitter* if s/he reports that they have stopped smoking at 4 weeks and his/her expired-air CO level is less than 10 ppm [28] when assessed 4 weeks after the designated quit date (minus 3 days or plus 14 days).

The definitions of service user outcomes (i.e. outcomes 3 to 5 above) were the same as those used for collection of routine statistics in the NHS smoking cessation service. A saliva sample was taken from service users at the end of the second session to measure 3OH cotinine and cotinine for biochemical verification of nicotine metabolic profile and for DNA extraction.

### Data collection

As a part of the contractual agreement, pharmacies providing smoking cessation services are required to collect user engagement and outcome data for monitoring and commissioning purposes. These routine administrative data were collected electronically using Sonar and Service Pact software; smoking cessation advisors enter these data on a regular basis. These data were then made available to the researchers by local authority public health commissioners for those pharmacies that participated in the STOP study. At the end of the pilot trial, anonymised data were extracted for all users enrolled in the service during the study period. Where users gave consent, pharmacists provided the client identification number which enabled access to the full set of routine data for that service user. Additional patient level data not available from the routine data set were collected using case report forms (CRFs).

Cluster level descriptive data were collected from consenting community pharmacies by the trial team using CRFs, including number of staff, number of smoking advisors and type of pharmacy. No baseline data were collected on pharmacies approached but not recruited.

Aggregated service user data (anonymised at the pharmacy level) were extracted from publicly available databases over the Internet, and stored on a secure database developed and maintained by the Pragmatic Clinical Trials Unit data management team. The information extracted included the area, general practice, gender, age in years, ethnicity and entitlement to free prescriptions.

### Sample size

As this was a pilot trial we did not undertake a formal sample size calculation. Based on our previous experience, to ensure that we had sufficient numbers to explore the feasibility and acceptability of the intervention, a sampling frame of 12 pharmacies and 96 service users was chosen; we anticipated the service user recruitment to be completed within 5 months. We planned to randomise twice as many pharmacies to the intervention group as to the usual practice group. We believed this to be a large enough sample to provide a reasonable understanding of likely impact of the intervention on throughput and to estimate the rates with reasonable precision (http://www.rds-london.nihr.ac.uk/How-to-design-a-study-find-funding/Statistics/sample-size-feasibility-study.aspx).

### Statistical methods

Data were analysed descriptively, without recourse to hypothesis testing. Continuous outcomes were summarised using mean, median and range values, with confidence intervals calculated to aid interpretation. Numbers and proportions were presented for categorical outcomes. Intracluster correlation coefficients (ICCs) were calculated using analysis of variance method (loneway command in Stata) for service user outcomes 2 and 3.

No interim analyses were undertaken. All analyses were carried out using Stata version 12.1.

## Results

**Fig. 1:**
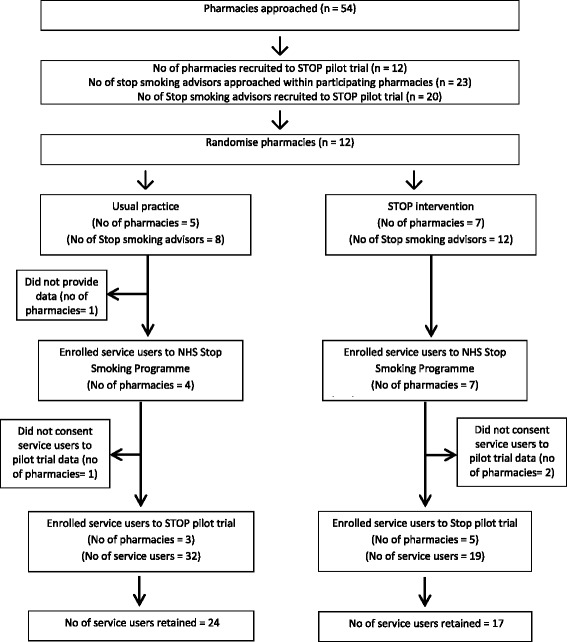
Recruitment flowchart of pharmacies and service users [34]

The pharmacy and service user throughput in STOP pilot trial is summarised in Fig. 1. We invited 54 community pharmacies and recruited 12 over 5 weeks from end January to March 2015. Of the 12 pharmacies recruited, 7 and 5 were randomised to the STOP intervention and usual practice, respectively. The majority (91.7%) were independent pharmacies (Table 1). Half of the pharmacies (*n* = 6) had only one trained smoking cessation advisor. The majority of advisors that we approached agreed to take part (20 of 23).

**Table 1:** Characteristics of pharmacies consented to take part in STOP pilot trial

Pharmacy characteristics	Allocation				Total	
	Usual practice		STOP intervention			
	No.	%	No.	%	No.	%
Total	5	100.0%	7	100.0%	12	100.0%
Average no of prescriptions per month						
4000 or less	1	20.0%	2	28.6%	3	25.0%
4001–6000	2	40.0%	1	14.3%	3	25.0%
6001–8000	0	0.0%	1	14.3%	1	8.3%
More than 8000	1	20.0%	0	0.0%	1	8.3%
Not provided	1	20.0%	3	42.9%	4	33.3%
Number of staff						
2	0	0.0%	1	14.3%	1	8.3%
3	0	0.0%	1	14.3%	1	8.3%
4	2	40.0%	2	28.6%	4	33.3%
5	2	40.0%	2	28.6%	4	33.3%
6	0	0.0%	1	14.3%	1	8.3%
11	1	20.0%	0	0.0%	1	8.3%
Number of STOP smoking advisors						
1	1	20.0%	5	71.4%	6	50.0%
2	2	40.0%	1	14.3%	3	25.0%
3	2	40.0%	0	0.0%	2	16.7%
5	0	0.0%	1	14.3%	1	8.3%
Type of pharmacy						
Independent pharmacy	4	80.0%	7	100.0%	11	91.7%
Chain pharmacy	1	20.0%	0	0.0%	1	8.3%

Service user recruitment began in March 2015 and was closed in August 2015 to work within the projected recruitment timelines. We were unable to obtain routine data from one pharmacy which was randomised to usual practice. Furthermore, two pharmacies in the intervention group and one in usual practice group did not obtain consent for using individual-level data from any service users. The final dataset included cluster level outcome data from 7 intervention and 4 control pharmacies and individual-level outcome data from 5 intervention and 3 control pharmacies respectively.

In total, 621 service users were enrolled in the service (i.e. throughput) with 302 in intervention and 319 in the usual practice arms. (Table 2). The average number of service users per pharmacy was higher in the usual practice arm (79.8, 95% CI: 19.0 to 140.5 vs 43.1, 95% CI: −4.3 to 90.5).

**Table 2:** Numbers of service users by pharmacy over the pilot study period

Pharmacy	Intervention	No of service users
C01	Usual practice	106
C02	Usual practice	63
N02	Usual practice	116
N04	Usual practice	34
^a^T01	Usual practice	–
C03	STOP intervention	48
C04	STOP intervention	11
C05	STOP intervention	42
N01	STOP intervention	154
N03	STOP intervention	25
N05	STOP intervention	14
N06	STOP intervention	8

^a^Did not provide data for analysis

Fifty-one of 621 service users (8.2%) consented to provide additional data on quit outcomes together with biological specimens for DNA extraction and calculation of nicotine metabolic rate (Table 3). The two arms tended to differ in baseline user characteristics. In the STOP intervention arm, the majority of participants were women (73.7% vs 43.8% in usual practice arm) and tended to be younger (median 42 vs 48.5 years in usual practice arm). Also a greater proportion of the intervention arm was entitled to free prescriptions (89.5% vs 81.3% in usual practice arm). The usual practice arm was more ethnically diverse than the STOP intervention arm.

**Table 3:** Characteristics of service users consented to provide additional data to STOP pilot trial

Service user characteristics	Allocation				Total	
	Usual practice		STOP intervention			
	No.	%	No.	%	No.	%
Total	32	100.0%	19	100.0%	51	100.0%
Area						
City and Hackney	8	25.0%	12	63.2%	20	39.2%
Newham	24	75.0%	7	36.8%	31	60.8%
Gender						
Female	14	43.8%	14	73.7%	28	54.9%
Male	18	56.3%	5	26.3%	23	45.1%
Age (in years)						
Mean (SD)	46.8 (11.7)		42.6 (9.5)		45.2 (11.0)	
Median (range)	48.5 (19 to 69)		42 (27 to 57)		48 (19 to 69)	
Entitlement to free prescriptions						
Yes	26	81.3%	17	89.5%	43	84.3%
No	6	18.8%	2	10.5%	8	15.7%
Ethnicity						
African	3	9.4%	1	5.3%	4	7.8%
Any other mixed/multiple ethnic background	2	6.3%	2	10.5%	4	7.8%
Any other white background	4	12.5%	3	15.8%	7	13.7%
Any other ethnic group	2	6.3%	0	0.0%	2	3.9%
Bangladeshi	2	6.3%	0	0.0%	2	3.9%
Caribbean	1	3.1%	0	0.0%	1	2.0%
English/Welsh/Scottish/Northern Irish/British	14	43.8%	12	63.2%	26	51.0%
Irish	2	6.3%	0	0.0%	2	3.9%
Pakistani	1	3.1%	0	0.0%	1	2.0%
Not stated	1	3.1%	1	5.3%	2	3.9%

Pilot results suggest that there is a possibility of higher rates of retention in the stop smoking service and quitting in consented service users in the STOP intervention arm. 17 of 19 service users were retained at 4 weeks in the STOP intervention arm (89.5%, 95%CI: 66.9% to 98.7%) and 24 of 32 (75.0%, 95%CI: 56.6% to 88.5%) in the usual practice arm. 10 of 19 in the STOP intervention arm (52.6%, 95%CI: 28.9% to 75.6%) and seven of 32 (21.9%, 95% CI: 9.3% to 40.0%) in the usual practice arm had stopped smoking, confirmed by exhaled carbon monoxide.

The intra cluster correlation coefficients for retention were 0.11 (95% CI: 0 to 0.50) in usual practice arm vs 0.05 (95% CI: 0 to 0.54) in intervention arm and for quit rates 0.29 (95% CI: 0 to 0.83) in usual practice arm vs 0.05 (95% CI: 0 to 0.53) in intervention arm. The overall smoking advisor and pharmacy participation rates were 87.0% (95% CI: 66.4% to 97.2%) and 22.2% (95% CI: 12.0% to 35.6%), respectively.

The service user consent rate to the study was slightly lower in the STOP intervention arm compared to the usual practice arm (6.3% vs 10.0%).

No adverse events were reported.

## Discussion

Expanding the role of community pharmacists in primary healthcare is a cornerstone of government policy and health promotion activities such as smoking cessation are fundamental to this expanded role. However, the optimum methods for delivering this new role are under-researched, and there have been few rigorous evaluations of complex interventions in this setting.

The STOP research programme seeks to fill this gap by developing an educational package for NHS smoking cessation advisors in community pharmacies. Our pilot results suggest that the STOP intervention may have the potential to increase the number of smokers retained within the NHS Smoking cessation service (89.5% vs 75.0%) and subsequently stop smoking (52.6% vs 21.9%); in any case, a further trial is warranted. In line with good practice, we have reported ICCs in this paper. However, our primary outcome in the main trial is number of smokers, a measure of throughput in the service. Hence, we have not revised our original sample size calculation for the main trial based on these ICC results.

We used simple randomisation which led to 7 pharmacies in the intervention and 5 in the usual practice arms, rather than the 2:1 ratio specified at the outset. As this was a pilot study aiming to assess the feasibility and acceptability of the STOP intervention, this caused no concerns for analysis.

Recruiting clinicians and patients to clinical trials is often problematic with the majority of trials failing to recruit to target and within time [29, 30]. Therefore, we assessed the likely participation rates of pharmacies and stop smoking advisors to enable accurate planning of the main trial. Our invitation to take part in the STOP study was well received by pharmacies and advisors. 21 of 54 pharmacies invited expressed an interest in taking part, and 14 were visited. We recruited the 12 pharmacies required from the 14 pharmacies visited, over a period of 5 weeks. This period was in line with the length of time we expected when planning the pilot. Twenty of the 23 advisors working within 12 recruited pharmacies agreed to take part. This response was reassuring and showed that recruitment to the main trial is feasible.

More importantly, the pilot was useful in providing practical insights on how best to conduct the definitive trial and shortcomings of our present logistical arrangements. The challenges that we encountered in conducting this pilot trial, for example understanding the complex organisational structure both between and within community pharmacies, are not specific to smoking cessation but will be generally relevant for other researchers in this clinical setting. How we resolved those issues could provide a template for future research, not just in smoking cessation and health behaviour change, but in a broad range of studies examining the wider role of the pharmacist. A more detailed collection of qualitative data with formal analysis would have been useful to explore organisational aspects relevant to implementation and evaluation of the intervention in community pharmacies.

### Implications for further development of the STOP intervention

There was little evidence that our intervention increased the numbers of smokers being engaged in the service. Whilst in this pilot trial we trained only smoking cessation advisors, counter assistants are the first point of contact with the customers, and thus particularly important in engaging smokers in the service. In the main trial therefore all staff including counter assistants will be specifically targeted. Training sessions for counter assistants may need to have a different content to those for cessation advisors. Also to accommodate the work schedules of counter assistants which are different from the advisors, training sessions need to be held at times suitable for them. Further, the turnover of counter staff may be higher than that for advisors, and new methods of providing training for replacement staff may need to be developed.

The intervention is currently designed so that session one is more basic and hence suitable for counter assistants. However, this lead to comments from some experienced advisors that the session was not immediately relevant to their work. We propose to split session one into a more detailed discussion relevant specifically to advisors and to develop a separate session aimed specifically at the needs of the counter assistants.

### Pharmacies as businesses

There is potential tension between the pharmacy as a *business* and pharmacy as *a healthcare provider*. Implications such as paying staff while they attend training and managing their cover, the ramifications for the pharmacy as a business (i.e. selling and providing other services) need to be fully considered. However, the smoking cessation service may in itself be a revenue stream for the pharmacy since payments are received for throughput and for successful quitters. Some pharmacists had recognised this and saw delivering an effective smoking cessation service as part of their business plan. The intervention might seek to encourage this view more generally.

Over the course of the trial, we came to understand the complex management structures in community pharmacies better, which have implications both for refining the intervention and for conducting the trial. Some pharmacies are functionally only *outlets* of small chains of two or three pharmacies; thus, involving the managers of individual outlets as well as the owners from the start of this study is important. We have now identified such chains from publicly available data and propose to use this information to target the intervention more effectively. These issues also need to be taken into account in determining the unit of allocation in the trial.

### Applicability of pilot trial methods and findings to future definitive trial

One of the aims of this pilot trial was to consider the practicalities of processes that we intend to use in the future definitive trial. The aspects we focused on were recruitment of pharmacies/ service users, identifying factors that may influence outcomes and data collection particularly around feasibility of using routine datasets.

Our first thoughts were to consent pharmacies as a unit, as they were the unit of randomisation. Through our preparatory work however it became clear that it is important to secure the support of individual advisors. Hence, we changed our consenting procedure to seek individual advisor as well as the pharmacy owners’ consent before randomisation. In contrast to higher than expected overall pharmacy and advisor recruitment rate, service user consent rates lagged behind those that we expected. We were aiming to recruit 96 service users, but only 51 were chosen to participate over the course of the pilot study. We found that obtaining written user consent at the end of the second session as we originally intended was not feasible in most cases. This was the main reason for poor user consent rates at the start of the pilot trial. Therefore, we revised the process to obtain consent at the end of the first session which did not intrude on the usual flow of the service. Although these rates improved substantially when we changed the recruitment process, we have revised the target service user recruitment rates for the definitive trial.

We plan to use routinely collected data for outcome assessment in the STOP definitive trial, therefore it was important for us to pilot the data collection process to ensure that we are fully aware of the pros and cons of using these data. As the pilot trial progressed, it became clear that taking part in research studies was new to pharmacy staff; they found consenting service users and using CRFs burdensome, which meant that some advisors did not consent service users to the trial and did not complete the CRFs as instructed. This highlighted the need for incorporating some additional research-specific training, specifically on gaining service user written consent, collecting saliva samples, documenting, anonymising, storing and transferring data to the study research team. Further, these workload considerations suggested a need to explore all avenues for accessing routinely collected data to reduce additional burden on advisors. Recent reorganisation of the NHS stop smoking service commissioning and NHS confidentiality guidelines, made the process of accessing routinely collected data arduous and time consuming. However, through the pilot trial we have streamlined the data extraction process for the main STOP trial which is now efficient and gives high quality data.

The NHS smoking cessation services collect cessation outcomes at 4 weeks, and therefore long-term follow-up data are not available from routine datasets. In addition, biochemical verification of cessation is not always undertaken routinely. We set a secondary outcome for the definitive trial to assess cessation at 4 weeks to align with the outcome used to measure quitting in the routine service. However, long-term data on cessation are important since they will allow us to compare the results of our intervention with other smoking cessation interventions in similar randomised controlled trials [31]. The minimum standard in randomised trials of smoking cessation intervention is biochemically verified cessation at 6 months [32]. Thus, in the definitive trial, we will ask for written user informed consent at the end of the first session for STOP study additional data collection for a six-month follow-up. In addition, longer term follow-up will allow more accurate estimation of quality adjusted life years gained and thus the incremental cost effectiveness of the STOP intervention over usual care.

The primary outcome of the definitive evaluation, which is a pragmatic cluster randomised trial will be recruitment (engagement) into the NHS smoking cessation services (i.e. throughput). This is collected from routine data at the cluster level under gate keeper agreement, therefore will not be affected by service user recruitment which is conducted after the smoker has been recruited to the service and is necessary only to collect data on biochemically validated long-term cessation.

We acknowledge the notable differences in service user characteristics across the two trial arms. When there are a small number of clusters available as in our study, there is relatively high probability of chance imbalance between treatment arms [33]. We believe the additional training on service user consenting, changes to the service user recruitment process and also the significant increase in sample size of the definitive trial would counteract these imbalances to some extent. Additionally we will identify those service user characteristics that have a significant bearing on cessation outcomes a priori and account for those in our analyses.

Another important aspect of this pilot trial was assessing the feasibility of collecting pharmacy level data at baseline to avoid any unintended imbalances in the main trial. From our preparatory work for the STOP research programme, pharmacy size and type of pharmacy were identified as important factors to take into account. Unlike some other healthcare services, pharmacy service users are not required to register with pharmacies. Hence, selecting an appropriate measure of pharmacy size was not straightforward. Following further discussions, we opted to collect average number of prescriptions per month as a measure of pharmacy size and footfall. However, it became clear that some pharmacists are reluctant to provide this information directly, therefore we revised our processes to access this information from publicly available sources.

## Conclusion

The pilot trial has been useful in resolving several of the uncertainties that we identified a priori when designing the trial. It also highlighted the need to train counter staff as well as stop smoking advisors. The solutions we have put in place are well thought through and drawn from our previous experiences in a number of different settings. We are confident that the main trial can proceed as planned.

## Abbreviations

CO: Carbon monoxide
CRFs: Case report forms
DNA: Deoxyribonucleic acid
ICC: Intracluster correlation coefficient
LFU4W: Lost to follow up at 4 weeks
NCSCT: National Centre for Smoking Cessation and Training
NHS: National Health Service
NIHR: National Institute for Health Research
NRES: National Research Ethics Service
REC: Research Ethics Committee
STOP: The Smoking Treatment Optimisation in Pharmacies
UKCRN: UK Clinical Research Network
